# PPAR*α* Agonist Fenofibrate Reduced the Secreting Load of *β*-Cells in Hypertriglyceridemia Patients with Normal Glucose Tolerance

**DOI:** 10.1155/2016/6232036

**Published:** 2016-02-29

**Authors:** Jia Liu, Rui Lu, Ying Wang, Yanjin Hu, Yumei Jia, Ning Yang, Jing Fu, Guang Wang

**Affiliations:** ^1^Department of Endocrinology, Beijing Chao-Yang Hospital, Capital Medical University, Beijing 100020, China; ^2^Department of Cardiology, Beijing No. 6 Hospital, Beijing 100007, China; ^3^Physical Examination Center, Beijing Chao-Yang Hospital, Capital Medical University, Beijing 100020, China

## Abstract

Hypertriglyceridemia is an important risk factor associated with insulin resistance and *β*-cell dysfunction. This study investigated the effects of hypertriglyceridemia and fenofibrate treatment on insulin sensitivity and *β*-cell function in subjects with normal glucose tolerance. A total of 1974 subjects with normal glucose tolerance were divided into the normal TG group (NTG group, *n* = 1302) and hypertriglyceridemia group (HTG group, *n* = 672). Next, 92 patients selected randomly from 672 patients with hypertriglyceridemia were assigned to a 24-week fenofibrate treatment. The HTG group had increased waist circumference (WC), body mass index (BMI), homeostasis model assessment of insulin resistance (HOMA-IR), and homeostasis model assessment of *β*-cell function (HOMA-*β*) and decreased high-density lipoprotein cholesterol (HDL-C) compared with the NTG group (all *P* < 0.01). The 24-week fenofibrate treatment significantly decreased the WC, BMI, TG, HOMA-IR, and HOMA-*β* levels and increased the HDL-C levels in the patients with hypertriglyceridemia (WC, BMI, and HOMA-IR: *P* < 0.05; TG, HDL-C, and HOMA-*β*: *P* < 0.01). The fenofibrate treatment significantly alleviated insulin resistance and reduced the secreting load of *β*-cells in the hypertriglyceridemia patients with normal glucose tolerance.

## 1. Introduction

Type 2 diabetes is a growing health issue due to its increased prevalence and lack of an ideal therapy [1]. Insulin resistance and *β*-cell dysfunction are considered the main pathophysiologic mechanisms of type 2 diabetes [2]. Epidemiological studies have demonstrated that hypertriglyceridemia is an important risk factor associated with insulin resistance and *β*-cell dysfunction [3, 4]. Lipoprotein lipase (LPL) gene knockout heterozygous mice, an animal model of genetic hypertriglyceridemia, exhibited significant insulin resistance, compensatory increased insulin secretion, and ultimately impaired glucose tolerance [5]. Therefore, it might be possible to prevent the development of type 2 diabetes mediated through alleviating insulin resistance and *β*-cell dysfunction by controlling hypertriglyceridemia.

Fenofibrate is a specific peroxisome proliferator-activated receptor *α* (PPAR*α*) agonist and is widely used as a triglyceride- (TG-) lowering agent [6]. Our previous study showed that fenofibrate increased tetrahydrobiopterin level and decreased production of reactive oxygen species through upregulating the level of intracellular guanosine 5′-triphosphate cyclohydrolase-I (GTPCH-I) in human umbilical vein endothelial cells [7]. Some large-scale clinical researches (FIELD and ACCORD) mainly focused on the cardiovascular benefits of fenofibrate treatment in patients with type 2 diabetes [8, 9]. Recently, many studies have shown some beneficial effects of fenofibrate on glucose metabolism in patients with prediabetes, type 2 diabetes, or metabolic syndrome [10, 11]. However, there is lack of clinical evidence supporting the effects of lowering TG for insulin sensitivity and *β*-cell function in hypertriglyceridemia patients with normal glucose tolerance. In this study, we aimed to investigate the effects of hypertriglyceridemia and fenofibrate treatment on insulin sensitivity and *β*-cell function in subjects with normal glucose tolerance.

## 2. Materials and Methods

### 2.1. Subjects

We enrolled 1974 subjects with normal glucose tolerance who had undergone a routine physical examination at the Beijing Chao-Yang Hospital affiliated to Capital Medical University from March 2012 to March 2014. Oral glucose tolerance tests (OGTT) and blood pressure measurements were performed at screening. Subjects with hypertension, prediabetes, diabetes, coronary artery disease, liver or renal function impairment, infectious disease, systemic inflammatory disease, or cancer were excluded. Subjects taking agents known to influence glucose or insulin metabolism and/or being treated with lipid-lowering drugs were also excluded. Hypertriglyceridemia was defined by a plasma TG level ≥1.7 mmol/L according to the guideline of NCEP ATP III and the Endocrine Society [12, 13]. Based on TG levels, all subjects were divided into the normal TG group (NTG group, *n* = 1302) and the hypertriglyceridemia group (HTG group, *n* = 672). All enrolled subjects provided a written informed consent. The protocol of this study was approved by the Ethics Committee of the Beijing Chao-Yang Hospital affiliated to Capital Medical University.

### 2.2. Interventional Study

This study is not a randomized, controlled trial. In the interventional study, 96 patients were randomly selected from 672 patients with hypertriglyceridemia. Four patients were excluded from participation because they were planning to become pregnant. All patients gave their informed written consent about the side effects of fenofibrate treatment. Finally, 92 patients with hypertriglyceridemia were assigned to 24 weeks of fenofibrate (200 mg/d). Only 83 patients were investigated after 24 weeks of fenofibrate treatment. The reasons for the 9 subjects not completing the study were incompliance and loss of contact. There were no changes in lifestyle interventions or medications during the study period. Anthropometric data and laboratory assays were performed three times: at baseline and after 12 and 24 weeks of fenofibrate administration.

### 2.3. Clinical and Biochemical Measurements

A standard questionnaire was used to collect the information about health status and medications. Waist circumference (WC) was measured on a horizontal plane at the level of the iliac crest with an anthropometric tape. Height and weight were measured to the nearest 0.1 cm and 0.1 kg, respectively, by the same trained group. Venous blood samples were obtained after overnight fasting. Plasma samples of all participants were stored at −80°C. High-density lipoprotein cholesterol (HDL-C), low-density lipoprotein cholesterol (LDL-C), TG, and total cholesterol (TC) were measured by colorimetric enzymatic assays using an autoanalyzer (Hitachi 7170). Fasting blood glucose (FBG) and fasting insulin (FINS) were measured at the central chemistry laboratory in Beijing Chao-Yang Hospital affiliated to Capital Medical University. Body mass index (BMI) was calculated as weight in kilograms divided by height in meters squared. According to the following formula, the homeostasis model assessment of insulin resistance (HOMA-IR) and that of *β*-cell function (HOMA-*β*) were calculated to test for insulin resistance and *β*-cell function, respectively: HOMA-IR = [FINS (mIU/L) *∗* FBG (mmol/l)/22.5] and HOMA-*β* = [20 *∗* FINS (mIU/L)/FBG (mmol/l) − 3.5] [14].

### 2.4. Statistical Analysis

Data were analyzed using SPSS 17.0 (SPSS, Inc., Chicago, IL). Continuous data were expressed as means ± SD. Because TG, FINS, HOMA-IR, and HOMA-*β* did not follow a normal distribution, the values were given as medians and upper and lower quartiles. After logarithmical transformation, the data of TG, FINS, HOMA-IR, and HOMA-*β* were fitted to a normal distribution for comparison. Differences between groups were analyzed by independent sample *t*-test and ANOVA test. Differences of proportions were analyzed by a chi-square test. Pearson, Spearman correlation, and covariance analyses were used. Changes in parameters from baseline values within a group were evaluated using a two-tailed paired *t*-test. Statistical significance was inferred when *P* < 0.05.

## 3. Results

### 3.1. Baseline Characteristics of the NTG and HTG Groups

The baseline characteristics of the NTG and HTG groups are summarized in Table 1. The two groups had similar sex ratios. The HTG group had higher age, WC, and BMI levels than the NGT group (age: 42.37 ± 10.38 versus 40.89 ± 11.99 years, *P* < 0.05; WC: 93.68 ± 6.62 versus 89.12 ± 5.42 cm, *P* < 0.01; BMI: 26.14 ± 3.85 versus 23.32 ± 4.40 kg/m^2^, *P* < 0.01). Increased plasma TC and LDL-C levels and decreased plasma HDL-C levels were observed in the HTG group compared to the NTG group (TC: 5.25 ± 0.90 versus 4.77 ± 0.85 mmol/L; LDL-C: 2.96 ± 0.74 versus 2.79 ± 0.72 mmol/L; HDL-C: 1.06 ± 0.21 versus 1.34 ± 0.30 mmol/L; all *P* < 0.01). The HTG group had significantly higher FBG, FINS, HOMA-IR, and HOMA-*β* than the NTG subjects [FBG: 5.47 ± 0.34 versus 5.33 ± 0.38 mmol/L; FINS: 16.17 (11.25–21.93) versus 9.80 (6.77–13.79) mIU/L; HOMA-IR: 3.86 (2.76–5.42) versus 2.30 (1.56–3.29); HOMA-*β*: 164.15 (114.05–228.80) versus 110.30 (77.02–154.92); all *P* < 0.01].

### 3.2. Correlation between Plasma TG and the Values of HOMA-IR and HOMA-*β*

**Figure 1:**
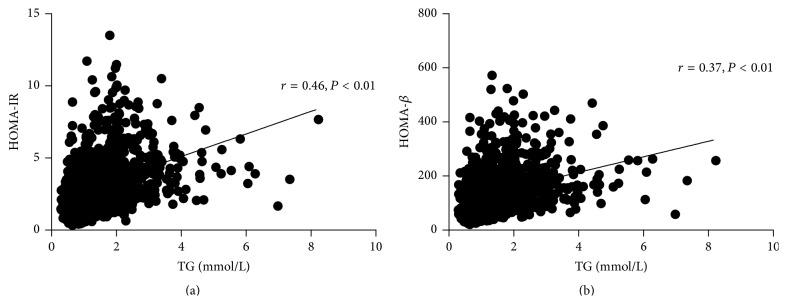
Correlation between plasma TG and the values of HOMA-IR (a) and HOMA-*β* (b). The plasma levels of TG were positively correlated with the values of HOMA-IR and HOMA-*β* in all the participants (HOMA-IR: *r* = 0.46, *P* < 0.01, 95% confidence interval 0.42 to 0.51; Figure 1(a)) (HOMA-*β*: *r* = 0.37, *P* < 0.01, 95% confidence interval 0.31 to 0.41; Figure 1(b)).

The plasma levels of TG were positively correlated with the values of HOMA-IR and HOMA-*β* in all the participants (HOMA-IR: *r* = 0.46, *P* < 0.01, 95% confidence interval 0.42 to 0.51; Figure 1(a)) (HOMA-*β*: *r* = 0.37, *P* < 0.01, 95% confidence interval 0.31 to 0.41; Figure 1(b)). These positive correlations were still observed after adjusting for age, WC, and BMI (HOMA-IR: *r* = 0.26, HOMA-*β*: *r* = 0.23, all *P* < 0.01).

Moreover, we still found a positive correlation between plasma levels of TG and HOMA-*β* in all the participants, after the adjustment for HOMA-IR (*r* = 0.06, *P* < 0.05).

### 3.3. Influence of Fenofibrate on the Anthropometric Parameters and Lipid Profile

The WC and BMI levels were significantly decreased from baseline at 24 weeks of the fenofibrate treatment (WC: from 93.00 ± 7.78 to 91.27 ± 7.59 cm; BMI: from 25.67 ± 3.11 versus 25.08 ± 3.35 kg/m^2^; all *P* < 0.05; Table 2). However, there were no significant changes in these parameters at 12 weeks. Compared with the baseline, fenofibrate decreased the TG levels and increased HDL-C after 12 and 24 weeks of treatment [TG: 12 weeks: from 2.70 (1.93–3.56) to 1.75 (1.47–2.00) mmol/L; 24 weeks: from 2.70 (1.93–3.56) to 1.63 (1.21–2.04) mmol/L; all *P* < 0.01] [HDL-C: 12 weeks: from 1.03 ± 0.18 to 1.29 ± 0.27 mmol/L, *P* < 0.05; 24 weeks: from 1.03 ± 0.18 to 1.40 ± 0.27 mmol/L, *P* < 0.01]. However, the fenofibrate treatment did not significantly affect TC or LDL-C at 12 or 24 weeks.

### 3.4. Influence of Fenofibrate on the Parameters of Glucose Metabolism

The fenofibrate treatment at 12 or 24 weeks did not significantly change the FBG levels (Table 2). Fenofibrate significantly decreased the FINS levels at 24 weeks compared with baseline but not at 12 weeks [12 weeks: from 14.40 (8.85–23.45) to 12.80 (8.90–17.10) mIU/L, *P* > 0.05; 24 weeks: from 14.40 (8.85–23.45) to 10.40 (7.10–15.40) mIU/L, *P* < 0.05]. At 12 and 24 weeks, HOMA-IR and HOMA-*β* were significantly decreased from baseline after the fenofibrate treatment [HOMA-IR: 12 weeks: from 3.26 (2.53–5.18) to 2.59 (1.98–3.85); 24 weeks: from 3.26 (2.53–5.18) to 2.42 (1.46–4.12); all *P* < 0.05] [HOMA-*β*: 12 weeks: from 124.31 (65.45–194.17) to 110.00 (45.38–131.58), *P* < 0.05; 24 weeks: from 124.31 (65.45–194.17) to 95.10 (48.75–123.07), *P* < 0.01]. However, fenofibrate treatment did not cause a significant decrease in HOMA-*β* at 12 or 24 weeks after adjustment for HOMA-IR.

## 4. Discussion

In the present study, the hypertriglyceridemia patients with normal glucose tolerance exhibited significant higher HOMA-IR and HOMA-*β* levels compared to the NTG group. The plasma levels of TG were positively correlated with the values of HOMA-IR and HOMA-*β* after adjusting for potential confounders. The fenofibrate treatment significantly decreased the plasma TG levels and the values of HOMA-IR and HOMA-*β* in the hypertriglyceridemia patients with normal glucose tolerance.

Several epidemiological studies have shown that hypertriglyceridemia is associated with insulin resistance and type 2 diabetes [3, 4]. Our study is consistent with these studies and demonstrated that the patients with hypertriglyceridemia exhibited significantly higher HOMA-IR levels than the control group [15]. The plasma TG levels were positively correlated with HOMA-IR after adjusting for age, WC, and BMI. The mechanism connecting hypertriglyceridemia to insulin resistance is still not fully understood. First, elevated plasma TG provokes ectopic lipid storage in insulin targeted organs, such as the liver and skeletal muscle, and induces the onset of insulin resistance by interrupting the insulin signaling pathway, activating oxidative stress and endoplasmic reticulum stress [5, 16, 17]. Moreover, increasing plasma TG levels caused a chronic inflammatory state by promoting hepatic expression and circulating levels of proinflammatory factors, such as tumor necrosis factor-*α* (TNF-*α*) and interleukin-1*β* (IL-1*β*) in a nuclear factor *κ*B-dependent pathway [16, 17]. In our study, the patients with hypertriglyceridemia had higher WC and BMI levels than the NTG subjects, which suggested that the patients with hypertriglyceridemia had obvious central obesity and more visceral adipose. In obese subjects, hypertrophic adipocytes and infiltration of inflammation cells in white adipose tissue cause dysfunction of adipose tissue and result in increased expression of proinflammatory factors and decreased adiponectin expression, eventually contributing to a chronic low-grade inflammatory state and insulin resistance [18].

Our study also demonstrated that the patients with hypertriglyceridemia exhibited significantly higher FINS and HOMA-*β*, which is similar to another recent study in nondiabetic subjects with hypertriglyceridemia [19]. These results might suggest a compensatory increase in insulin secretion and a higher secreting load of islet *β*-cells in patients with hypertriglyceridemia. In our study, the participants were at the state of normal glucose tolerance, and their islet *β*-cells were still in the compensatory state. However, if this is sustained, chronic overload of islet *β*-cells will contribute to deterioration of *β*-cell function that accompanies the development of type 2 diabetes [20, 21]. Moreover, long-term elevated plasma lipids induce *β*-cell apoptosis and impair insulin secretion mediated by deposition of fatty acyl-CoA derivatives in islet *β*-cells [22, 23].

Type 2 diabetes is a growing health issue due to its increased prevalence and lack of an ideal therapy [1], and how to prevent type 2 diabetes is gradually becoming an important hotspot issue. Recently, several prospective trials demonstrated that a large proportion of prediabetic patients developed type 2 diabetes even after a lifestyle intervention and/or metformin treatment [24, 25]. The animal model of genetic hypertriglyceridemia exhibited significant insulin resistance and compensatory increased insulin secretion and developed impaired glucose tolerance ultimately [5], which suggested that the insulin resistance and chronic overload of islet *β*-cells already existed before prediabetes happens. The present study showed that, in the hypertriglyceridemia patients with normal glucose tolerance, fenofibrate treatment significantly attenuated the increased insulin resistance and secreting load of islet *β*-cells. Therefore, fenofibrate might be available to prevent the development of prediabetes and type 2 diabetes by improving insulin sensitivity and decreasing the secreting load of *β*-cells in hypertriglyceridemia patients with normal glucose tolerance. The exact mechanism involved in the beneficial effects of fenofibrate on insulin resistance and *β*-cell function has not been fully elucidated. Our study showed that fenofibrate treatment did not cause a significant decrease in HOMA-*β* after adjustment for HOMA-IR, which might suggest that fenofibrate reduces the secretory requirements for *β*-cells mainly by lowering insulin resistance. Some recent studies have shown that the PPAR*α* agonist significantly reduced muscle triglycerides and long-chain ester acyl coenzyme A accumulation, total liver triglyceride content, and visceral fat weight in high fat-fed male Wistar rats, which suggested the effects of the PPAR*α* agonist on reducing ectopic lipid storage [26, 27]. In addition, increased insulin sensitivity was also connected to reduced body weight [6]. In the present study, the fenofibrate treatment significantly decreased the WC and BMI of the patients with hypertriglyceridemia. Consistent results have also been shown in hypertriglycemic patients and high fat-fed animal models in other studies [11, 28]. The fenofibrate treatment led to weight loss effect mediated through stimulating mitochondrial biogenesis, thermogenesis, and fatty acid oxidation in a peroxisome proliferator-activated receptor gamma coactivator-1*α*-dependent pathway [6]. A recent study in diet-induced obese mice showed that fenofibrate significantly triggered browning of white adipocytes through stimulating irisin expression and uncoupling protein-1 transcription and led to increased energy consumption [6]. In addition, the fenofibrate treatment also decreased adipocyte size and moderated adipose tissue dysfunction in high fat diet-induced obese mice [29, 30]. In patients with impaired fasting glucose, a fenofibrate-based treatment moderated adipose tissue dysfunction and chronic inflammatory state by increasing adiponectin levels and decreasing resistin and interleukin-6 (IL-6) levels [31]. Furthermore, in our study, the fenofibrate treatment significantly increased HDL-C levels, which might also have beneficial effects on insulin resistance and *β*-cell dysfunction [32–34]. HDL stimulated the phosphorylation of AMP-activated protein kinase (AMPK), increased glucose uptake of myocytes, and contributed to improved insulin sensitivity [32, 33]. HDL-C also regulated *β*-cell function by removing cholesterol from *β*-cells [34].

It is worth mentioning that bezafibrate, another drug of fibrates, also displayed some beneficial effects on insulin resistance and glucose metabolism in patients with type 2 diabetes [35]. Different from fenofibrate, bezafibrate is a pan PPAR agonist and activates three PPAR subtypes including PPAR*α*, PPAR*δ*, and PPAR*γ* [36]. The activation of PPAR*γ* improves insulin sensitivity by upregulating adipogenesis and decreasing free fatty acid levels; meanwhile, the activation of PPAR*δ* correlates with enhancement of fatty acid oxidation and adaptive thermogenesis [36]. Although the additional activation of PPAR*γ* and PPAR*δ* might suggest a greater improvement in glucose and lipid metabolism, the effect on PPAR*γ* may be related to a higher risk of water retention, weight gain, and peripheral edema [36].

The present study has several limitations. First, our study was not a randomized, controlled trial, and it might introduce some bias. Then, our study estimated *β*-cell function and insulin resistance by HOMA-*β* and HOMA-IR, instead of the precise methods such as the hyperglycaemic and hyperinsulinemic clamp technique. It should also be recalled that HOMA-*β* is a surrogate of insulin secretion and thus does not directly measure the secreting load. Further randomized-controlled researches using the glucose clamp technique will likely be required to definitively confirm the beneficial effects of fenofibrate we are reporting in the present study. Last, one should acknowledge that long-term follow-up will be necessary to evaluate whether the fenofibrate treatment delays disease progression ultimately.

## 5. Conclusion

The elevated plasma TG is associated with insulin resistance and increased insulin secretion of *β*-cells. The fenofibrate treatment significantly alleviated insulin resistance and reduced the secreting load of *β*-cells in the hypertriglyceridemia patients with normal glucose tolerance. Therefore, fenofibrate might be available to prevent the development of prediabetes and type 2 diabetes by improving insulin sensitivity and decreasing the secreting load of *β*-cells in hypertriglyceridemia patients with normal glucose tolerance.

## Figures and Tables

**Table 1:** Baseline characteristics of the NTG and HTG groups.

Parameters	NTG group (*n* = 1302)	HTG group (*n* = 672)
Age, y	40.89 ± 11.99	42.37 ± 10.38^*∗*^
Gender, males/females	632/670	374/298
WC, cm	89.12 ± 5.42	93.68 ± 6.62^*∗∗*^
BMI, kg/m^2^	23.32 ± 4.40	26.14 ± 3.85^*∗∗*^
TC, mmol/L	4.77 ± 0.85	5.25 ± 0.90^*∗∗*^
LDL-C, mmol/L	2.79 ± 0.72	2.96 ± 0.74^*∗∗*^
HDL-C, mmol/L	1.34 ± 0.30	1.06 ± 0.21^*∗∗*^
TG, mmol/L	0.95 (0.71–1.26)	2.24 (1.95–2.88)^*∗∗*^
FBG, mmol/L	5.33 ± 0.38	5.47 ± 0.34^*∗∗*^
FINS, mIU/L	9.80 (6.77–13.79)	16.17 (11.25–21.93)^*∗∗*^
HOMA-IR	2.30 (1.56–3.29)	3.86 (2.76–5.42)^*∗∗*^
HOMA-*β*	110.30 (77.02–154.92)	164.15 (114.05–228.80)^*∗∗*^

Data are means ± SD unless indicated otherwise. TG, FINS, HOMA-IR, and HOMA-*β* are shown as medians and upper and lower quartiles. WC: waist circumference; BMI: body mass index; TC: total cholesterol; LDL-C: low-density lipoprotein cholesterol; HDL-C: high-density lipoprotein cholesterol; TG: triglyceride; FBG: fasting blood glucose; FINS: fasting insulin; HOMA-IR: homeostasis model assessment of insulin resistance; HOMA-*β*: homeostasis model assessment of *β*-cell function. ^*∗*^Significantly different at *P* < 0.05 versus control; ^*∗∗*^significantly different at *P* < 0.01 versus control.

**Table 2:** Comparison of clinical parameters after fenofibrate treatment for 12 and 24 weeks in patients with hypertriglyceridemia (*n* = 83).

Parameters	Baseline	12 weeks	24 weeks
Age, y	44.68 ± 10.51		
Gender, M/F	50/32		
WC, cm	93.00 ± 7.78	92.32 ± 7.26	91.27 ± 7.59^*∗*^
BMI, kg/m^2^	25.67 ± 3.11	25.29 ± 3.44	25.08 ± 3.35^*∗*^
TC, mmol/L	5.34 ± 0.68	5.11 ± 0.94	5.12 ± 0.66
LDL-C, mmol/L	2.79 ± 0.75	3.10 ± 0.72	3.02 ± 0.61
HDL-C, mmol/L	1.03 ± 0.18	1.29 ± 0.27^*∗*^	1.40 ± 0.27^*∗∗*^
TG, mmol/L	2.70 (1.93–3.56)	1.75 (1.47–2.00)^*∗∗*^	1.63 (1.21–2.04)^*∗∗*^
FBG, mmol/L	5.43 ± 0.49	5.45 ± 0.50	5.33 ± 0.46
FINS, mIU/L	14.40 (8.85–23.45)	12.80 (8.90–17.10)	10.40 (7.10–15.40)^*∗*^
HOMA-IR	3.26 (2.53–5.18)	2.59 (1.98–3.85)^*∗*^	2.42 (1.46–4.12)^*∗*^
HOMA-*β*	124.31 (65.45–194.17)	110.00 (45.38–131.58)^*∗*^	95.10 (48.75–123.07)^*∗∗*^

Data are means ± SD unless indicated otherwise. TG, FINS, hsCRP, HOMA-IR, and HOMA-*β* are shown as medians and upper and lower quartiles. WC: waist circumference; BMI: body mass index; TC: total cholesterol; LDL-C: low-density lipoprotein cholesterol; HDL-C: high-density lipoprotein cholesterol; TG: triglyceride; FBG: fasting blood glucose; FINS: fasting insulin; HOMA-IR: homeostasis model assessment of insulin resistance; HOMA-*β*: homeostasis model assessment of *β*-cell function. ^*∗*^Significantly different at *P* < 0.05 versus baseline; ^*∗∗*^significantly different at *P* < 0.01 versus baseline.
